# Impact of Viral Decontamination Method on Cytokine Profile of COVID-19 Patients

**DOI:** 10.3390/biomedicines9101287

**Published:** 2021-09-22

**Authors:** Davide Magrì, Anna Navarro, Federica Bergami, Elena Percivalle, Alessandro Ferrari, Teresa Lettieri, Luigi Calzolai, Antonio Piralla, Fausto Baldanti, Sabrina Gioria

**Affiliations:** 1 European Commission, Joint Research Centre (JRC), 21027 Ispra, Italy; davide.magri@ec.europa.eu (D.M.); Anna.NAVARRO-CUENCA@ec.europa.eu (A.N.); Teresa.LETTIERI@ec.europa.eu (T.L.); Luigi.CALZOLAI@ec.europa.eu (L.C.); 2 Molecular Virology Unit, Microbiology and Virology Department, Fondazione IRCCS Policlinico San Matteo, 27100 Pavia, Italy; federica.bergami01@universitadipavia.it (F.B.); e.percivalle@smatteo.pv.it (E.P.); alessandro.ferrari04@universitadipavia.it (A.F.); a.piralla@smatteo.pv.it (A.P.); fausto.baldanti@unipv.it (F.B.); 3 Department of Clinical, Surgical, Diagnostic and Pediatric Sciences, University of Pavia, 27100 Pavia, Italy

**Keywords:** safety assessment, SARS-CoV2, UVC, ultrafiltration device, BAL, inflammation, cytokines, chemokines

## Abstract

COVID-19 related morbidity and mortality have been often attributed to an exaggerated immune response. The role of cytokines and chemokines in COVID-19 and their contributions to illness severity are known, and thus their profiling from patient bronchoalveolar lavage (BAL) samples would help in understanding the disease progression. To date, limited studies have been performed on COVID-19 BAL samples, as the manipulation of such specimens (potentially containing live viruses) requires several laboratorial precautions, such as personnel training and special equipment, a requirement that not all laboratories can fulfil. Here, we assessed two fast and easily applicable methods (ultrafiltration and ultraviolet–C irradiation) for their impact on viral load removal or inactivation, respectively and on cytokine profiles preservation. Eight samples of BAL fluids from SARS-CoV2 patients with high viral load were tested. For both methods, complete removal was confirmed by lack of viral replication in Vero E6 cells and by RT-qPCR. Although both methods showed to remove completely the active SARS-CoV2 viral load, only UVC treatment has little or no quantitative effect on total cytokines/chemokines measurements, however cytokines profile and relative ratios are preserved or minimally altered when compared data obtained by the two different decontamination methods. Sample preparation and manipulation can greatly affect the analytical results; therefore, understanding if changes occurred after sample processing is of outmost importance for reliable data and can be useful to improve clinical practice.

## 1. Introduction

Although respiratory failure is the leading cause of death in COVID-19 patients, a broad spectrum of patient symptoms [1] and systemic complications resulting from an overactive host immune response have been extensively described [2]. Host cells utilize cytokines and chemokines to coordinate nearly all aspects of an immunogenic response to pathogens: acute inflammation, innate response, and adaptive immunity. Indeed, evidence increasingly confirms that cytokine release syndrome (CRS, or cytokine storm), an exuberant, hyperinflammatory immune response, is responsible for severe COVID-19 complications, including acute respiratory distress syndrome (ARDS), multi-organ failure, and death [3,4,5]. Therefore, the study of the pathogenesis of SARS-CoV-2 cannot be limited only to the analysis of non-respiratory samples, such as blood, but should include, when available, the bronchoalveolar lavage (BAL) specimens, as the lungs are the main site of the severe infection. Studying the underlying inflammatory response in these samples can help identifying new biomarkers of inflammation that both indicate the severity of COVID-19 and allow distinguishing it from severe influenza. It could also offer insights for new therapies. 

If from one hand it is crucial to assess cytokine profile in BAL [6], the stringent requirements for handling of these samples reduce opportunities to perform immunological assays on these specimens, unless there is a complete removal of the active viral load. Working with high-hazard pathogens generally requires containment level 3 or 4 (BSL3 or BSL4), which implies following strict safety and regulatory standards [7]. Since the outbreak of the SARS-CoV-2 pandemic, the need to quickly process huge amounts of contaminated specimens has led to a necessary rethinking of laboratory methods to streamline analytical work while ensuring the safety of operators. Virus inactivation has previously been established using several methods such as heat treatment, use of formalin, Triton X-100, β-propiolactone, γ-ray irradiation and N_2_ gas plasma [8,9,10,11,12]. While these methods have been shown to inactivate partially or effectively viruses, they can alter the structure of proteins and nucleic acids molecules, thus potentially modifying protein analytes of interest such as cytokines rendering the sample useless for diagnostic purposes. The ability to simultaneously measure multiple analytes with reduced volume and workload is particularly attractive for immunological studies on clinical samples and the Luminex measurement platform is extensively used due to the low limit of detection and reliability [13], even though this technology is definitely not a practicable solution if employed in a BSL3 or BSL4 environment. In this regard, Dowall et al. have developed a protocol for Luminex analysis of hazardous pathogenic samples in BSL2, based on formalin treatment to inactivate viruses preserving the functionality of the protein components [13]. However, this approach might still not be applicable in all laboratories, as the methodology, based on several incubation and washing steps still requires the use of ad-hoc instruments difficult to handle in BSL of class higher than two. In this study, we evaluated the efficiency of the active viral load removal from BAL samples of COVID-19 patients using ultrafiltration or UVC irradiation, considered as two fast and easy applicable methods. In addition to previous work [14,15], mainly limited in evaluating the efficacy of the decontamination methods, here we also assessed on the treated samples if cytokines and chemokines were still preserved and measurable. Moreover, comparison of cytokine profiling is presented.

Although much effort has been spent into the processing/storage of cytokines from blood samples, there is limited and dated knowledge of the influence of sample preparation on the recovery of cytokines from non-blood samples such as BAL [16]. This study underlies the importance of the critical evaluation of the changes undergone by the analytes of interest during the processing in order to produce reliable and useful data for the identification of new biomarkers, such as cytokine levels, correlated to the clinical status of the patient also in non-conventional specimens and anatomical compartments.

## 2. Materials and Methods

### 2.1. Samples

BAL samples were collected from SARS-CoV-2-positive patients within 48–72 h of admission to the intensive care unit at the Fondazione IRCCS Policlinico San Matteo of Pavia (Pavia, Italy). 

The presence of SARS-CoV-2 RNA was assessed using specific real-time RT-PCR as previously described [17,18]. Samples were anonymized before the analysis, and no clinical information were available, apart from cycle threshold (Ct) values.

### 2.2. Virus Removal Procedures

#### 2.2.1. Ultrafiltration Method

Ultrafiltration method was applied for the removal of the virus load from the BAL samples. For this purpose, Amicon^®^ Ultra-100 (nominal pore size 10 nm) centrifugal filter device (cat UFC 510024, Merck Millipore Ltd., Milan, Italy), were used. A volume of 500 µL of each BAL samples was loaded in the microcentrifuge tube, which was centrifuged at 3000× *g* for 30 min at 4 °C. The filtrate was collected from the sample reservoir with a pipette and transferred into a 1.5 mL eppendorf tube and stored at −80 °C till further use. The work was handled in the biosafety level 3 laboratory of the IRCCS Policlinico San Matteo of Pavia (Pavia, Italy).

#### 2.2.2. UVC Irradiation Method

The UVC device used was optimized and calibrated to enable accurate and controlled UVC treatment of test samples. A collimated beam setup was fabricated based on a dual chamber construction. The top chamber contains the UVC light source, the electronic driver, and a shutter system to control the exposure times of the samples while keeping the lamp output stable. Samples treatment have been performed according to Storm et al. [15]. The irradiance level of the lamp inside the treatment chamber was measured using a calibrated UVC sensor system (Spectroradiometer GL Optic Spectis 5.0 Touch with detector GL Opti Probe 5.1.50), which provided irradiance patterns and levels. UVC exposure was performed by irradiation with UVC (254 nm) of 500 µL of BAL samples in UVette tubes (Eppendorf, Milan, Italy) for 1 h (enough to inactivate up to 99% of SARS-CoV-2 [19]). The UV light source (UV-4 S/L, order no. 2950440, Herolab, Wiesloch, Germany) was placed at a distance of 3 cm above the bottom of the tube. The emitted light intensity was UVC (254 nm) = 1940 µW/cm^2^ at a distance of 3 cm, as measured by radio-metric analysis. This corresponds to an applied light dose of 1.94 mJ/cm^2^ per second, while µW = 10^6^ J/s. UVC treated samples were transferred into a 1.5 mL eppendorf tube and stored at −80 °C till further use. All UV experiments on BAL samples were performed in the biosafety level 3 laboratory of the San Matteo of Pavia (Pavia, Italy).

### 2.3. Evaluation of Viral Load Removal

#### 2.3.1. RT-qPCR

Complete removal of the viral load was also assessed by Reverse Transcription quantitative PCR (RT-qPCR) assay performed on the treated samples. EURM-019, a synthetic RNA sequence reference material (RM) containing the 72 bp amplicon for the nucleocapsid 3 (N3) gene(EURM-019, European Commission’s Joint Research Centre (JRC)) [20]. The RM was used as a positive control to verify the correct transcription and amplification during the SARS-CoV-2 detection by RT-qPCR. The pCoV2 plasmid, a standard pUC57 vector containing a synthetic cDNA sequence of RM EURM-019, was used to produce the standard curve. A ten-fold serial dilution of the pCoV2 plasmid (5 × 10^5^ to 5 copies/reaction) was used to generate the standard curve with the log10-linear regression of triplicate Cycle threshold (Ct) values. The detection of N3 gene by RT-qPCR was performed in 25 µL reaction mixtures using RNA UltraSense™ One-Step Quantitative RT-PCR System (Thermo Fisher Scientific, Waltham, MA, USA). The RT-qPCR mixture contained 5 μL of 5× reaction Mix, 1.25 μL Enzyme Mix, 200 nM of forward primer (5′- GGG AGC CTT GAA TAC ACC AAA A -3′), 200 nM of reverse primer (5′- TGT AGC ACG ATT GCA GCA TTG -3′), 200 nM of TaqMan probe (5′-FAM- AYC ACA TTG GCA CCC GCA ATC CTG -QSY-3′) [21,22], and 7.5 μL of sample. The RT-qPCR assays Applied Biosystems 7900HT Real Time PCR Systems with ABI 7900 software SD2.4 (Thermo Fisher Scientific, Waltham, MA, USA) using automatic settings for threshold and baseline. Thermal cycling conditions consisted of RT at 50 °C for 15 min, denaturation and Taq polymerase activation at 95 °C for 2 min, and 45 cycles of 95 °C for 15 s followed by 58 °C for 30 s. RT-qPCR reactions were performed in triplicates for each sample and the sample Ct mean was used for further analyses. Samples were always analyzed with corresponding positive (RM) and negative controls (DNase-and RNase-free water).

#### 2.3.2. Confirmation of Virus Inactivation by Virus Isolation

To investigate the infectious potential of BAL, virus isolation was attempted with the Vero E6 (VERO C1008 (Vero 76, clone E6, Vero E6); ATCC^®^ CRL-1586™) cell line. In detail, a 200 µL sample was inoculated onto a Vero E6 confluent 24 well microplate for virus isolation. After 1 h of incubation at 33 °C in 5% CO_2_ in air, the inoculum was discarded and 1 mL of medium for respiratory viruses was added (Eagle’s modified minimum essential medium supplemented with 1% penicillin, streptomycin and glutamine, and 5 mg/mL trypsin) to each well. Cells were incubated at 33 °C in 5% CO_2_ in air and observed by light microscopy every day for cytopathic effect. After a 7-day incubation, 200 µL of supernatant was used for molecular investigation [18].

### 2.4. Comparison Immunogenicity on Decontaminated Samples

The 8-plex quality control mixture provided in the Bio-Plex Pro™ Human Cytokine 8-plex Assay (cat. M50000007A) has been diluted 1:1 in sterile PBS (Gibco, Thermo Fisher Scientific, Waltham, MA, USA). A volume of 60 µL has been taken for each of the following condition: UVC exposure for 10 and 60 mins as described in Section 2.2.2 or applied to Amicon^®^ Ultra-100 centrifugal filter device (Cat UFC510096, Merck Millipore Ltd., Burlington, MA, USA) untreated or pre-treated with BSA (1 mg/mL) (Cat A4506, Sigma-Aldrich, Milan, Italy) or PEG600 (1 mg/mL) (Cat 81180 Fisher Scientific, Milan, Italy). After centrifugation at 3000 g for 30 min at 4 °C, the supernatant was collected from the sample reservoir with a pipette. After removal of any liquid left in the column, a volume of 60 µL has been applied to the filter for washing of the filter itself and then collected. All samples were run on the Bio-Plex200 System and on the 2100 Bioanalyzer as described in Section 2.5 and Section 2.6, respectively. Three independent experiments have been performed. 

### 2.5. Bioanalyzer

The protein electrophoresis analysis was performed with the 2100 Bioanalyzer using the High Sensitivity Protein 250 kit assays, to evaluate the size and to quantify the amount of the protein samples. Each sample was equilibrated using the Standard labelling buffer and labelled with the fluorescent dye. The samples were diluted 200 times in milliQ-water and denatured at 95 °C for 5 min in reducing condition by adding 3.5 Vol.-% of 1 M Dithiothreitol (DTT) solution to the sample buffer. The samples were cooled and loaded on the microfluidic chip for the electrophoresis, according to the manufacture instructions. All reagents and instrument were from Agilent Technology, Santa Clara, CA, USA.

### 2.6. Multiplex Immunoassays for Cytokine Detection 

Samples prepared by ultrafiltration or UVC exposure were thawed on ice and tested for the presence of cytokines using commercial ELISA-based microarrays that simultaneously measure multiple proteins in a single sample aliquot. Multiplex Bio-Plex Pro™ Human Cytokine 8-plex Assay (cat. M50000007A) was used for assessing the production of IL-2, IL-4, IL-6, IL-8, IL-10, TNF-α, IFN-γ, and GM-CSF. Singleplex for IL-1β (Cat 171B5001M) and IL-1Ra (Cat 171B5002M) were also included. Samples were run according to the manufacturer’s instructions. An internal calibrator was used. Cytokines were analysed with the Bio-Plex200 System using the Bio-Plex Manager™ software, and data were analysed by the Bio-Plex Data Pro^TM^ software, using five-parametric curve fitting. For each cytokine, assay ranges and LOD were provided by the manufacturer. All reagents and instruments, including Washing Station and Shaker Incubator, were from BIO-RAD Laboratories (Segrate, Italy).

## 3. Results 

### 3.1. Viral Load Removal

The performance on SARS-CoV-2 virus load removal by ultrafiltration or UVC irradiation methods from eight BAL specimens of COVID-19 affected patients has been assessed. In order to evaluate the non-infectivity of the treated BAL specimens, BAL samples were inoculated onto Vero E6 cells and tested for the presence of SARS-CoV-2 RNA by specific RT-qPCR. Original BAL samples had a median Ct value of 20.9 (range 17.8–24.2) and all showed cytopathogenic effect (CPE) within 48 h after inoculation. No CPE was observed in cells inoculated with ultrafiltration or UVC treated samples at 5 and 7 days of incubation. Considering the initial high SARS-CoV-2 load, according to La Scola et al [23], if the virus had not been efficiently removed by the decontamination process it should have led to virus isolation in 90–95% of the cases.

As reported in Table 1, there is no detectable viral RNA in all samples treated with ultrafiltration, confirming the absence of viral RNA. Whereas, for the UVC treated samples a very low signal of amplification was found in few samples at quite high Ct values, meaning a presence of very low amount of SARS-CoV-2 RNA. However, as for diagnostic, the presence of RNA fragments does not imply the presence of any replicative virus [24]. Based on the data obtained, it can be concluded that active viral load removal was successfully achieved by ultrafiltration devices and UVC irradiation. 

### 3.2. Cytokine Recovery

**Figure 1:**
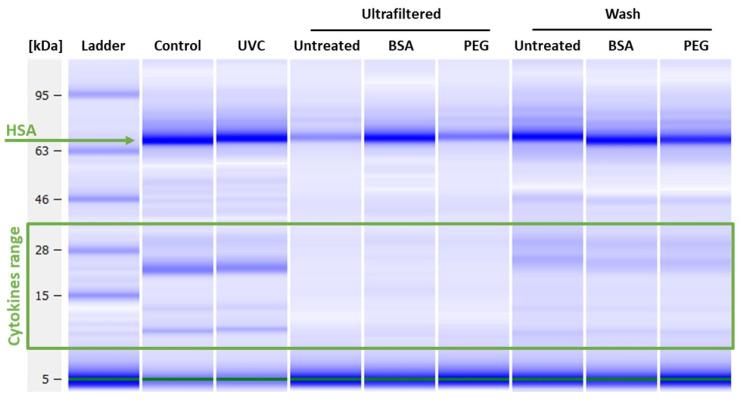
Digital electrophoresis of the viral samples decontaminated with UVC irradiation or ultrafiltration using 100 kDa filters, or filters pre-treated with BSA, or PEG600. Following the centrifugation step, the filters have been washed with PBS and the collected washing solution was analysed (Wash).

Before testing the effect of the decontamination methods on cytokine measurements of patient BAL samples, we firstly characterized and verified quantitatively the effect of both treatments on the analytes of interest. To do so, we considered an ad-hoc prepared cytokines/chemokines control mixture. Untreated control mixture was divided in aliquots that were subject to ultrafiltration or to UVC treatment. We characterized the impact on the overall concentration of cytokines/chemokines through digital electrophoresis (Figure 1). The analysis has been performed also on samples treated using BSA and PEG600 pre-conditioned filters, to avoid unspecific binding to the filter, as well as on the analyte collected from the filter washing after the ultrafiltration.

**Figure 2:**
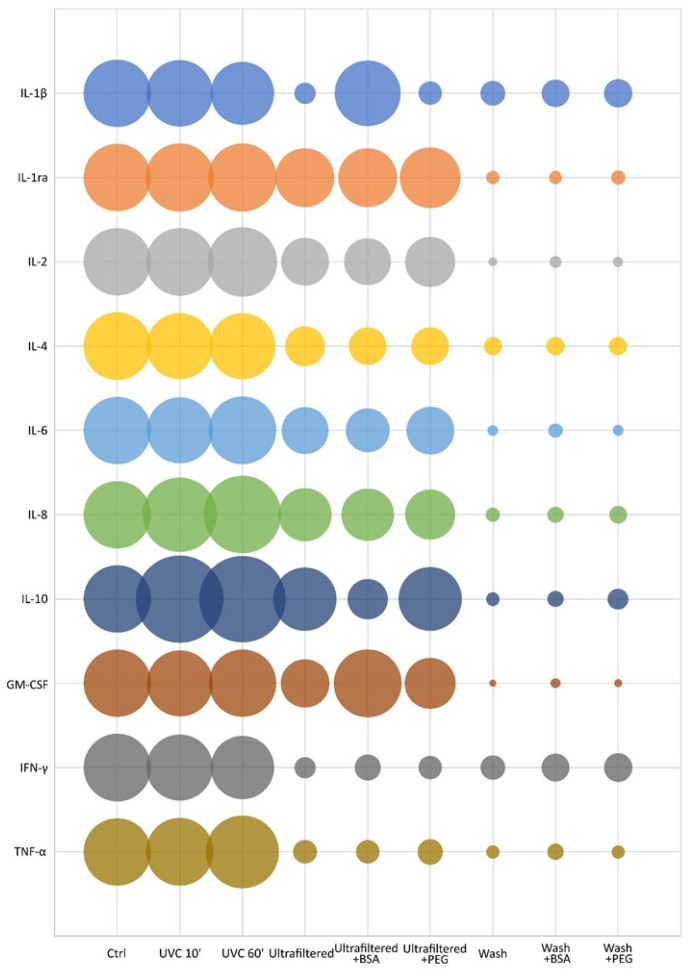
Effects of the viral decontamination methods (ultrafiltration using 100 kDa columns or UVC irradiation) on the recovery of several cytokines and chemokines expressed as % compared to the 100 % of the untreated control mixture (the area of the bubble represents the amount of cytokine in percentage). On the vertical axis the cytokines analysed, on the horizontal axis the different treatment conditions. Results are the average of three independent experiments (CV < 0.4).

The analysis shows that UVC treated samples have an overall better recovery than those treated by ultrafiltration. Part of the loss in the ultrafiltered samples is due to the non-passing volume remaining in the filter even after prolonged centrifugation. Pre-conditioning with BSA or PEG600 the filter resulted in a slight improvement of the overall recovery (5 and 1.5 folds respectively), however still too low. Bands in the range of the cytokines molecular weights are observable in the sample washing, meaning that an abundant part of proteins did not pass through the pores of the filter remaining loosely attached to it. By evaluating the total amount of recovered proteins in the various fractions, and also considering the non-passing volume, it appears evident an important loss of cytokines, most probably due to the interactions and binding with the filter membrane, as already observed in other studies [25]. To further confirm the observations revealed by digital electrophoresis, assessing the specific recovery of every protein, the same samples were run on multiplex immunoassays for cytokine detection. Figure 2 reports the effects of both viral decontamination methods on the measurements of the group of cytokines and chemokines considered. Data are expressed as % of recovery compared to the untreated control mixture. 

The first evidence is that UVC treated samples show a complete recovery of the cytokines, while the samples prepared by ultrafiltration using untreated filters show a substantial recovery loss. Moreover, the data show no substantial improvement in the performance of the filters upon pre-conditioning, although a slight increase was observed with the use of BSA probably due to the partial passivation of the negative charge of the cellulose filter matrix, which favours the passage of cytokines with lowest isoelectric point (IL-1β and CM-CSF). Another interesting aspect is observable in the UVC treated samples, where IL-8, IL-10, and to a lesser extent of TNF-α, show a quantitative increase of recovery compared to the control. This increment should be due to the disassembly of the dimeric forms of IL-8 and IL-10, and trimeric form of TNF-α, respectively, induced by the UVC exposure. In addition, no significant changes on the recovery of the cytokines and chemokines considered were observed between UVC treatment for 10 min or 1 h.

**Figure 3:**
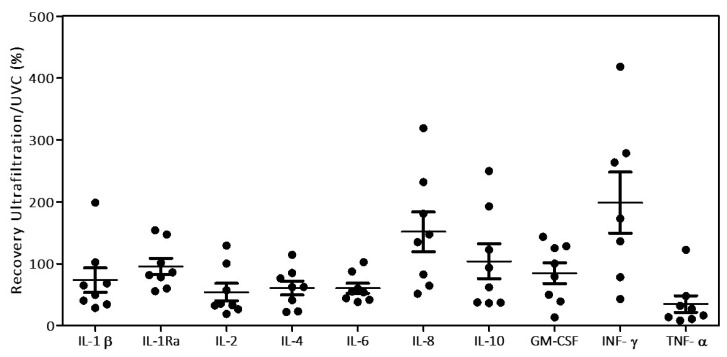
Effects of the virus decontamination methods (using UVC irradiation and AMICON^®^-100 kDa ultrafiltration) on the recovery of several cytokines and chemokines from BAL samples. Distribution plot indicates the cytokine/chemokines recovery upon treatments, expressed as ultrafiltration/UVC treated ratio in percentage (black spots), average (black horizontal dash) and the standard errors are reported for each analyte.

Both ultrafiltration and UVC exposure approaches have been applied to the eight BAL samples and their effect on cytokines and chemokines measurements have been evaluated by using multiplex immunoassays for cytokine detection. Results of protein recovery are reported in Figure 3.

Despite the high variability in the recovery, it is visible that only in few cases (IL-8 and INF-γ) samples obtained by ultrafiltration method recover better than UVC-treated samples, thus partially confirming what observed in the previous results with the cytokines control mixture. The 100 kDa filter membrane used, with a cut off of 10 nm, should well retains SARS-CoV-2 virus having the size of circa 100 nm in diameter [26] while it was expected that cytokines and chemokines, in the size range of 10–40 kDa (<5 nm in diameter) would pass through according to the manufacturer’s specification.

### 3.3. Cytokine Profiling

**Figure 4:**
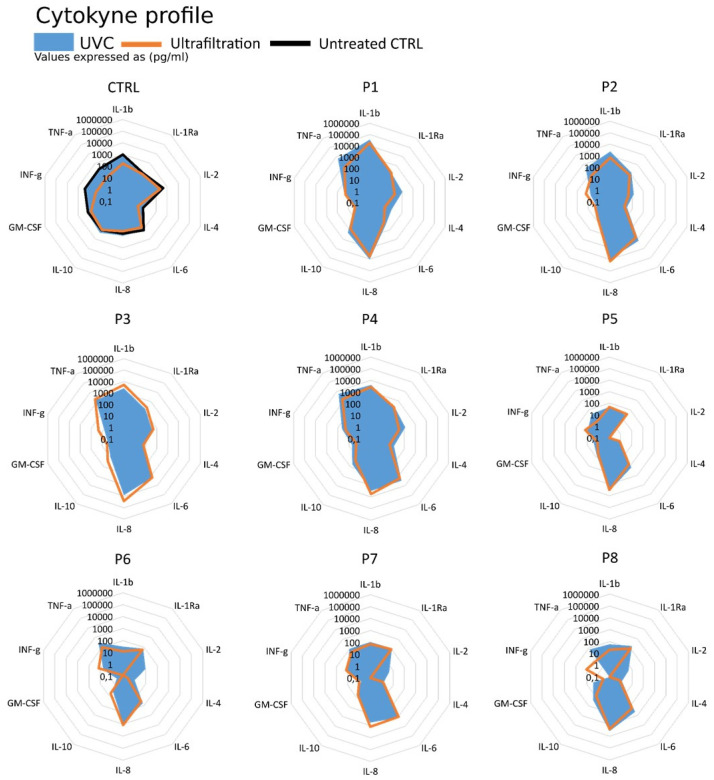
Cytokine/chemokine profiles of the patient BAL samples. The standard calibration mix has been used as reference control (CTRL) for the treatments.

Although the two decontamination methods result in completely different sample recovery, when analysing the overall group of target-proteins the preservation of cytokines and chemokines profiles become visible and the relative cytokine ratios appear conserved or only minimal altered (Figure 4).

The panel shown in Figure 4 highlights that the concentration profiles expressed in logarithmic scale appear unique for each sample analysed and specifically related to the specific pathological and inflammatory condition of every patient. Although processed with different decontamination methods, the two profiles (ultrafiltration or UVC treated) can be easily superimposed returning the same overall view on the modulators of the inflammatory and immune response. 

## 4. Discussion

The role of cytokines and chemokines and their contribution to the severity of illness in COVID-19 patients is now well recognized. Studying their expression in BAL samples could help to elucidate the physiopathology of the disease, identify new inflammation biomarkers linked to the severity of the illness, as well as offer insights into new therapies. Unfortunately, the stringent requirements needed for safe handling of these specimens reduce the opportunities for researchers to perform immunological assays on them unless measures are firstly taken to completely remove the active viral load. In this study, two methods for SARS-CoV2 active viral load removal from BAL samples are described and compared for their applicability in cytokines measurements as downstream application. The first method considered for virus removal was based on centrifugal filter de-vices, which has several advantages to make it applicable within a BSL3. Among others, the rapidity of use and the fact that the main equipment required is limited to a centrifuge, allowing several samples to be processed simultaneously. In addition, several volume sizes are possible based on the column volume selection. To date, as virus isolation is critical in research, vaccine production, and diagnostic workflows, centrifugal ultrafiltration devices with regenerated cellulose membranes have been generally used to achieve high titer virus stocks by concentrating virions and virus solutions [27]. In principle, to retain viral particles, the molecular weight cut-off of the filter membrane needs to be smaller than the particle (~2 times smaller than the molecular weight of the viral particle), but large enough to allow smaller components to filter through. Therefore, after appropriate selection of the cut-off of the filter, this principle could be considered for its potential to trap the virus in the filter, allowing the filtrate to be virus-free. For this work, we selected ultrafiltration column with a cut-off of 100 kDa, corresponding to a pore size of 10 nm, whereas SARS-CoV-2, as many other viruses, has a size of around 100-150 nm [26]. The second method considered for removal of the active viral load was by UVC irradiation, which was performed according to Storm et al. [15]. This method has several advantages: it is cheap, rapid to use and is applicable in BSL class 3 laboratories, although it might require, at least initially, some sophisticated instruments to set and control the radiation. Regarding the first method, our results show that by selecting an appropriate molecular weight cut-off (100 kDa), we were able to successfully use centrifugal ultrafiltration to remove the viral load from highly pathogenic BAL material while for the second method, UVC irradiation, we confirmed that it was able to effectively inactivate the virus. Although the chosen cut-off of the selected ultrafiltration membrane should not affect the passage of smaller protein components (such as interleukins) through the filter, when performing multiplex immunoassays for cytokine detection on the recovered samples we observed a consistent loss due to filter retention of cytokines and chemokines. Attempts to reduce the loss by pre-conditioning the filters with BSA or PEG600 did not substantially improve the recovery. The use of a UVC treatment under the condition described in this study was able to deactivate the virus while having little or no effect on the recovery of cytokines/chemokines. Although, it was noted that for cytokines normally present in the form of dimers or trimers, the action of the UVC treatment causes their dissociation resulting in a higher recovery. 

In conclusion, the UVC treatment represents a successful means of decontaminating SARS-CoV2 samples, allowing samples to be safely transported and used in laboratories, which may lack the proper biocontainment facilities necessary for handling potentially pathogenic viral strains. It should be noted that, although a reduced total recovery rate was observed when using the ultrafiltration method, both methods have shown similar cytokines ratio and profiling, thus offering the possibility for an evaluation of the overall inflammatory/immunological condition of the patients. The work has been performed on a limited number of samples as the aim was to evaluate the influence of sample preparation on the recovery and integrity of cytokines/chemokines from BAL samples so to produce reliable and useful data. In addition, information as patients’ data were not available.

In order to monitor the course of the disease to prevent the onset of serious inflammatory symptoms or to assess the impact of pharmacological treatments, both decontamination methods can be a useful support to give access to the study of such samples in BSL-2 laboratories. Both methods, in addition to being efficient in the removal of viral particles, have interesting characteristics such as easy implementation even in BSL-3 laboratories requiring simple and commonly available equipment, and allow a low waste of time and money (Table 2).

## 5. Conclusions

In this study, we presented two methods to recover cytokines and effectively eliminate the presence of active SARS-CoV-2 viruses from contaminated BAL samples: separation by centrifugal ultrafiltration and deactivation by UVC irradiation. Although the purposed methods can successfully remove the viral load, both processes partially affect the recovery of cytokines/chemokines. In particular, the interactions of the analytes with the filter material in ultrafiltration cause a loss for some of the cytokines of interest while the structural perturbation induced by UVC exposure causes the disassembly and increase in concentration of others. This opens new possibilities for researchers to study immune responses in the laboratory without the risk of exposure to the live virus. Studying cytokines in BAL samples could play a pivotal role in monitoring lung diseases such as SARS-CoV-2. To do this, research laboratories will increasingly require cost-effective and efficient methods to handle such samples safely while preserving biological information. Despite the quantitative effects shown, the cytokine profile of individual patient-derived samples does not undergo serious alterations. These results not only demonstrate the efficacy of simple methods in the treatment of virus-contaminated samples but underline the importance of awareness of the effect of the preparation on complex biological samples and their application in clinical diagnostics. The advent of the omics approach in biomedicine paves the way to new possibilities for researchers to study complex systemic physiological mechanism such as the immune response. The study of cytokines in BAL samples could play a fundamental role in monitoring lung diseases such as SARS-CoV-2 but it is essential to develop a network of laboratories capable of handling and studying these biological samples safely. Therefore, the development of increasingly economical, versatile, and efficient methods to manage such samples while preserving biological information will be indispensable.

## Figures and Tables

**Table 1:** PCR Amplification cycle threshold (Ct) values obtained from the N3 gene amplification of BAL specimens from SARS-CoV-2 positive patients treated with ultrafiltration or UVC. UND = Undetectable.

Sample	1	2	3	4	5	6	7	8
Ct	17.4	22.4	24.2	21.8	18.8	21.2	20.5	19.6
Filtered	UND	UND	UND	UND	UND	UND	UND	UND
UVC	28.17 ± 0.83	UND	UND	UND	33.92 ± 2.37	UND	UND	32.80 ± 1.08

**Table 2:** Comparison of the two decontamination techniques (by virus removal by ultrafiltration [28] or UVC inactivation) used in this study.

Advantages	Ultrafiltration	UVC Irradiation
Cheap	Yes	Yes
Rapid	Yes	Yes
Applicable for large volume	Up to 70 mL/time	Yes
Applicable in BSL3	Yes (only centrifuge required)	Yes (although might be difficult to set and control radiation)
Complete and efficient active virus removal	Yes	Yes
Recovery	Poor. Sample interacts with the filter causing sample loss	Good. Disassembly of the dimeric/trimeric forms of IL-8, IL-10 and TNF-α
Cytokine profiling	Yes	Yes

## Data Availability

The datasets for this study can be found at the following link: https://data.jrc.ec.europa.eu/collection/id-00252.
